# Characterization of early and late transition states of the folding pathway of a SH2 domain

**DOI:** 10.1002/pro.4332

**Published:** 2022-05-17

**Authors:** Angelo Toto, Francesca Malagrinò, Caterina Nardella, Valeria Pennacchietti, Livia Pagano, Daniele Santorelli, Awa Diop, Stefano Gianni

**Affiliations:** ^1^ Istituto Pasteur ‐ Fondazione Cenci Bolognetti, Dipartimento di Scienze Biochimiche “A. Rossi Fanelli” and Istituto di Biologia e Patologia Molecolari del CNR Sapienza Università di Roma Rome Italy

**Keywords:** intermediate, kinetics, mutagenesis, Φ‐value analysis

## Abstract

Albeit SH2 domains are abundant protein–protein interaction modules with fundamental roles in the regulation of several physiological and molecular pathways in the cell, the available information about the determinants of their thermodynamic stability and folding properties are still very limited. In this work, we provide a quantitative characterization of the folding pathway of the C‐terminal SH2 domain of SHP2, conducted through a combination of site‐directed mutagenesis and kinetic (un)folding experiments (Φ‐value analysis). The energetic profile of the folding reaction of the C‐SH2 domain is described by a three‐state mechanism characterized by the presence of two transition states and a high‐energy intermediate. The production of 29 site‐directed variants allowed us to calculate the degree of native‐like interactions occurring in the early and late events of the folding reaction. Data analysis highlights the presence of a hydrophobic folding nucleus surrounded by a lower degree of structure in the early events of folding, further consolidated as the reaction proceeds towards the native state. Interestingly, residues physically located in the functional region of the domain reported unusual Φ‐values, a hallmark of the presence of transient misfolding. We compared our results with previous ones obtained for the N‐terminal SH2 domain of SHP2. Notably, a conserved complex folding mechanism implying the presence of a folding intermediate arise from comparison, and the relative stability of such intermediate appears to be highly sequence dependent. Data are discussed under the light of previous works on SH2 domains.

## 1 INTRODUCTION

The protein folding problem is one of the central themes of biochemistry and molecular biology. An unfolded protein must undergo a myriad of conformational rearrangements to properly fold to the native state, however, from an experimental perspective, small globular proteins often show an intrinsic ability to self‐assemble in their native state in a two‐state fashion, without populating intermediate states along the folding pathway, with only native and denatured states that are accessible to the protein to be populated.^1^ Intermediate states, when present, are generally very limited in number and particularly elusive from an experimental detection and characterization, mostly because of their inherent transient nature.

Determining the folding mechanism of a protein relies in pinpointing the presence of intermediates and transition states along the reaction pathway and in characterizing their degree of structure.^2^, ^3^, ^4^, ^5^ Transition states, by definition, never accumulate, so that they must be characterized indirectly. A powerful strategy widely used to reach this goal is the Φ‐value analysis, a methodology based on a combination of mutagenesis and kinetics, which compares and normalizes the effect of single mutations in transition state with native state and allows to map the interactions occurring in the transition state(s) of the reaction.^6^ The strategy is also feasible to structurally characterize intermediate states. Intermediates can escape detection in classical kinetic (un)folding experiments, and their presence along the folding reaction can be inferred with different strategies. For example, non‐linear dependences of the logarithm of observed rate constants of (un)folding experiments as function of denaturant concentrations (i.e., chevron plots) is a well‐recognized signature of the existence of folding intermediates,^7^, ^8^ albeit data interpretation is not usually straightforward and rigorous analysis of different kinetic parameters must be taken into account.^9^

In previous studies we characterized the folding mechanism of the N‐terminal SH2 domain of SHP2, an important protein phosphatase with key role in several molecular pathways in the cell. Our results were compatible with a three‐state folding scenario, accounting for the presence of a low‐energy transiently populated on‐pathway intermediate revealed by a pronounced roll‐over effect in the refolding arm of the chevron plot. By performing a Φ‐value analysis we were able to characterize the structure of the intermediate and second transition state at nearly atomic resolution. Interestingly, our data showed rather high Φ‐values for the early intermediate state which were compatible with a surprisingly highly native‐like structure, further increased and consolidated in the late transition state.^10^

A powerful strategy to infer the mechanism of folding of a given protein relies in comparing its folding properties with proteins belonging to the same family, that is, sharing an overall same topology but different primary structures.^3^, ^11^, ^12^ In a recent work, we demonstrated the C‐SH2 domain to fold through a three‐state mechanism characterized by the presence of a high‐energy intermediate.^13^ In this paper, we report a Φ‐value analysis of the C‐terminal SH2 domain of SHP2. By performing an extensive site‐directed mutagenesis and a kinetic analysis we were able to characterize the structure of the early and late transition states of the folding reaction. Overall, the analysis of kinetic and thermodynamic data put in evidence a nucleation‐condensation mechanism of folding, with the presence of a structured folding nucleus surrounded by a lower degree of native‐like interactions, and subsequent locking in place in the late events of the reactions. Our results are discussed in comparison with what previously observed for the folding of the N‐SH2 domain of SHP2 and under the light of general folding properties of SH2 domains.

## 2 RESULTS

### 2.1 Φ‐value and Linear Free Energy Relationship analysis of the C‐SH2 domain of SHP2

In a recent work, by employing a combination of equilibrium and kinetic (un)folding experiments conducted at different experimental conditions, we analyzed the folding mechanism of the C‐SH2 domain of SHP2.^13^ An analysis of initial and final fluorescence of the unfolding reaction of the wild‐type C‐SH2 domain as function of [UREA] showed the dependence of the initial fluorescence (which resembles the signal of native protein) to be linear, denoting the absence of burst‐phase unfolding events. This aspect allowed us to conclude that the roll‐over in the unfolding arm of the chevron plot could be ascribable to a folding mechanism implying a change in the rate‐limiting step at high denaturant concentrations, with the presence of two transition states along the reaction pathway, and a high‐energy intermediate.

To infer the structural features of the early and late transition states of the C‐SH2 folding reaction we resorted to conduct a Φ‐value analysis. The Φ‐value analysis is a technique based on a combination of kinetic and extensive mutagenesis, which allows to determine the role of single aminoacidic residues in the folding of a given protein. By producing a number of site‐directed variants based on conservative truncation of residues lateral chains (Val to Ala, Leu to Ala, Ile to Val, Thr to Ser), that is, variants that may have a destabilizing effect on the protein without disrupting its native conformation,^6^, ^14^ and by monitoring the effect of such mutations on the thermodynamics of transition states and native state, it is possible to determine the degree of native‐like contacts which are formed by that residue in the transition states. These contacts are then reported as a normalization value (namely the Φ‐value) which measures the change in free energy in the transition state and normalizes it to the change in free energy between the ground states. As a result, Φ‐value tends to 1 if the mutated residue experiences a native‐like environment and therefore is fully “structured” in the probed state as it is in the native state, and it tends to 0 if it is “unstructured” as it is in the denatured state.

**FIGURE 1:**
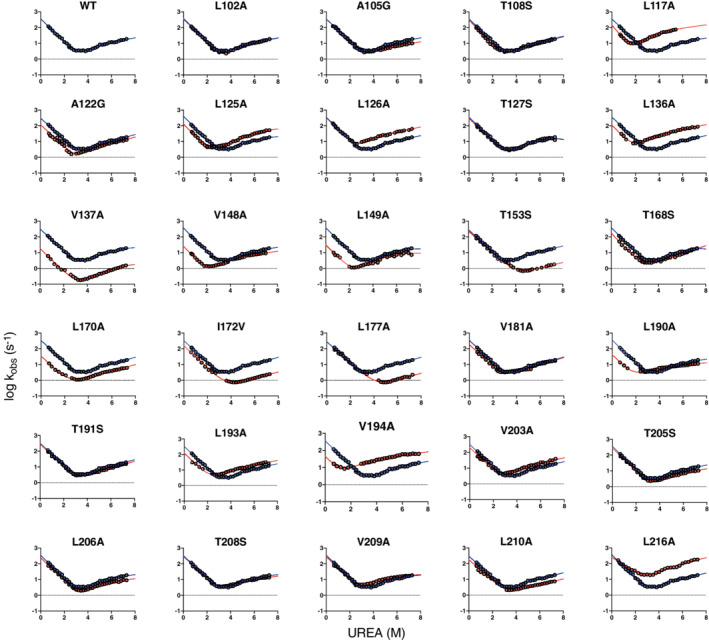
Chevron plots of C‐SH2 wt (in dark blue) and its site‐directed variants (in red). All the experiments were carried out in buffer TrisHCl 50 mM pH 8.0, at 25°C. As detailed in the text, data were globally fitted with an equation describing a three‐state folding mechanism with the presence of a high‐energy intermediate along the reaction pathway, and sharing kinetic m‐values for all data sets.

By following the general rules of Φ‐value analysis we designed 32 variants of the C‐SH2 domain (reported in Table 1). Of the 32 designed variants, three were too poorly expressed and could not be characterized. Experiments were performed by stopped‐flow methodology, monitoring the change in intrinsic fluorescence of the protein as function of time, by an 11‐fold dilution of the protein in buffer containing urea as denaturing agent for unfolding experiments, and protein in urea versus buffer at different final urea concentrations for refolding experiments. Buffer used was TrisHCl 50 mM, DTT 2 mM, pH 8.0 and experiments were performed at 25°C. In all the experiments conducted, the unfolding and refolding time courses were satisfactorily fitted with a single exponential equation. The dependences of the logarithm of the observed rate constants (*k*_obs_) as function of the concentration of denaturant (chevron plots) of all the variants explored are reported in Figure 1. In analogy to our previous work on the C‐SH2 domain,^13^ chevron plots displayed a deviation from linearity in their unfolding arm and were analyzed by using an equation taking into account the presence of a high‐energy intermediate along the reaction pathway

$$k_{\mathrm{obs}}=k_{f}^{0}\exp\left(-m_{f}\left[\text{UREA}\right]/\mathrm{RT}\right)+\frac{k_{u}^{0}\exp\left(m_{u}\left[\text{UREA}\right]/\mathrm{RT}\right)}{1+K_{\text{part}}\exp\left(m_{\text{part}}\left[\text{UREA}\right]/\mathrm{RT}\right)}$$

**FIGURE 2:**
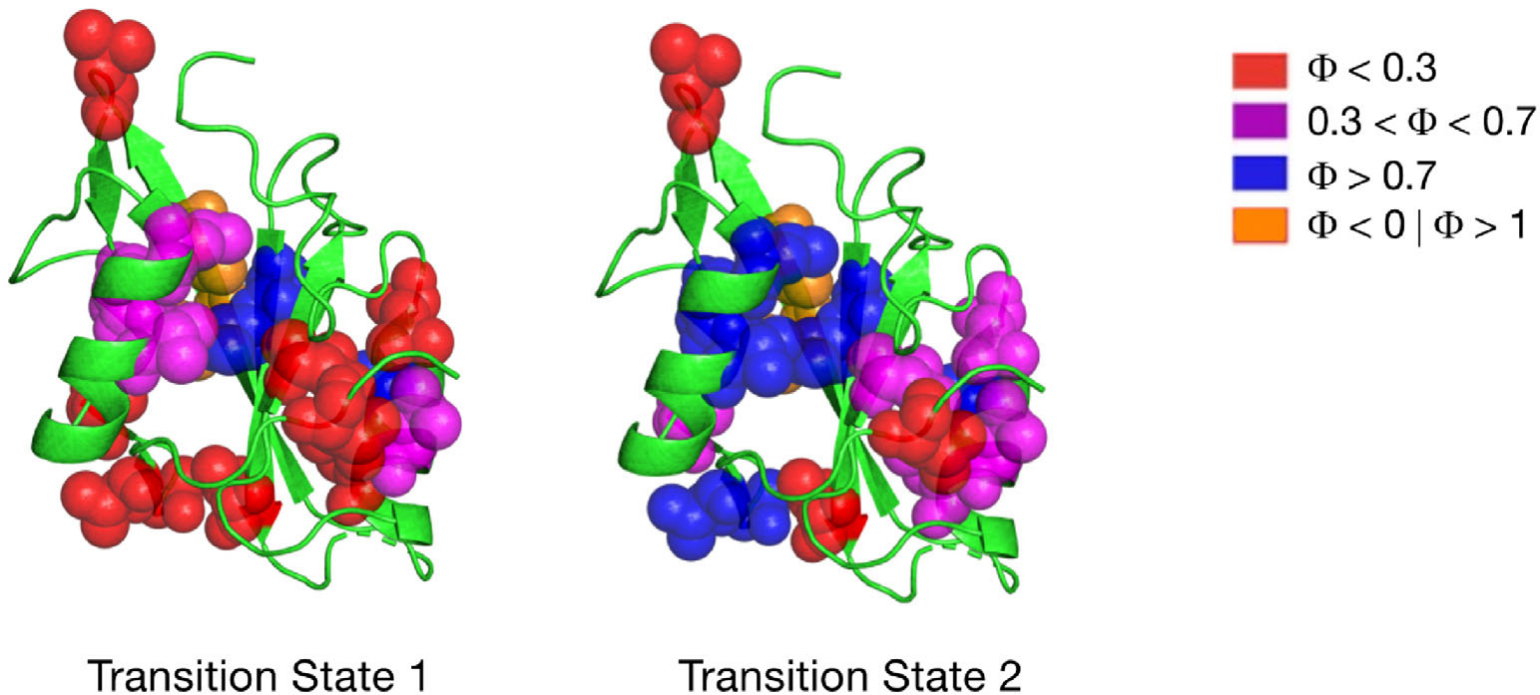
Distribution of Φ values calculated for the early (left) and late (right) transition states on the structure of the C‐SH2 domain. A generally accepted convention was followed, dividing the Φ values in four groups: low Φ values (Φ < 0.3) highlighted in red, intermediate Φ values (0.3 < Φ < 0.7) in magenta, high Φ values (0.7 < Φ < 1) in blue and unusual Φ values (Φ > 1 | Φ < 0) in orange.

To obtain a more reliable measurement of the kinetic parameters, all the chevron plots were analyzed by sharing kinetic m‐values.^15^ According to the model used to analyze kinetic data, we could calculate thermodynamic parameters for two energetic barriers obtaining two Φ‐values, one for the early transition state (Φ_TS1_) and one for the late transition state (Φ_TS2_). Following a generally accepted approach, to infer the structure of the two transition states we divided Φ‐values in four categories, high Φ‐values (0.7 < Φ < 1), intermediate Φ‐values (0.3 < Φ < 0.7), low Φ‐values (Φ < 0.3), and non‐canonical Φ‐values (i.e., below 0 or higher than 1), and mapped them on the three‐dimensional structure of the C‐SH2 domain following a well‐defined color‐code (Figure 2). Inspection of Figure 2a indicates the presence of a folding nucleus highlighted by high Φ‐values of residues V148 and L149, surrounded by a lower degree of native‐like structure, with the prevalence of low and intermediate Φ‐values in the early transition states. As the folding reaction proceeds, the degree of native‐like contacts increases, as depicted in Figure 2b. In fact, in the late transition state the folding nucleus appears to be more consolidated and surrounded by higher Φ‐values. Interestingly, inspection of Figure 2 reports anomalous Φ‐values (i.e., Φ > 1 and Φ < 0) in correspondence to residues V170 and I172, in both early and late events of folding. Non‐canonical Φ‐values may indicate that those residues are involved in non‐native interactions and can represent transient misfolding on the path to the native state.

**TABLE 1:** Folding and thermodynamic parameters of C‐SH2 domain variants.

	k_f_ (s^−1^)	k_u_ (s^−1^)	k_part_	∆∆G_TS1_ (kcal mol^−1^)	∆∆G_TS2_ (kcal mol^−1^)	∆∆G_eq_ (kcal mol^−1^)	Φ_TS1_	Φ_TS2_
Wt	300 ± 13	0.06 ± 0.01	0.017 ± 0.003					
L102A	280 ± 12	0.05 ± 0.01	0.013 ± 0.003	−0.13 ± 0.02	0.02 ± 0.01	−0.10 ± 0.06	*	*
A105G	330 ± 14	0.07 ± 0.02	0.036 ± 0.008	0.06 ± 0.02	−0.38 ± 0.07	0.01 ± 0.06	*	*
T108S	200 ± 9	0.07 ± 0.02	0.022 ± 0.004	0.11 ± 0.02	−0.05 ± 0.01	0.36 ± 0.06	*	*
L117A	90 ± 6	0.96 ± 0.14	0.037 ± 0.006	1.65 ± 0.15	1.20 ± 0.20	2.40 ± 0.40	0.31 ± 0.13	0.50 ± 0.12
A122G	110 ± 4	0.04 ± 0.01	0.018 ± 0.003	−0.16 ± 0.02	−0.21 ± 0.04	0.42 ± 0.08	1.38 ± 0.07	1.51 ± 0.09
L125A	100 ± 5	0.17 ± 0.03	0.018 ± 0.002	0.61 ± 0.03	0.60 ± 0.10	1.20 ± 0.20	0.50 ± 0.03	0.53 ± 0.13
L126A	280 ± 13	0.34 ± 0.06	0.031 ± 0.006	1.03 ± 0.06	0.70 ± 0.10	1.10 ± 0.20	0.04 ± 0.08	0.37 ± 0.08
T127S	260 ± 13	0.06 ± 0.01	0.017 ± 0.003	0.01 ± 0.02	0.01 ± 0.02	0.10 ± 0.02	*	*
L136A	90 ± 7	0.68 ± 0.10	0.045 ± 0.008	1.45 ± 0.11	0.90 ± 0.20	2.10 ± 0.40	0.32 ± 0.05	0.60 ± 0.08
V137A	20 ± 1	0.003 ± 0.001	0.012 ± 0.002	−1.72 ± 0.12	−1.50 ± 0.30	−0.02 ± 0.01	*	*
V148A	20 ± 1	0.08 ± 0.01	0.043 ± 0.008	0.19 ± 0.02	−0.36 ± 0.07	1.70 ± 0.30	0.89 ± 0.05	1.21 ± 0.04
L149A	28 ± 2	0.06 ± 0.01	0.028 ± 0.005	−0.04 ± 0.02	−0.34 ± 0.07	1.40 ± 0.30	1.00 ± 0.10	1.25 ± 0.05
T153S	260 ± 13	0.01 ± 0.01	0.070 ± 0.030	−0.73 ± 0.02	−1.50 ± 0.30	−0.70 ± 0.10	−0.13 ± 0.17	−1.40 ± 0.60
T168S	140 ± 7	0.05 ± 0.01	0.017 ± 0.003	−0.12 ± 0.02	−0.14 ± 0.03	0.34 ± 0.06	*	*
V170A	40 ± 2	0.04 ± 0.01	0.040 ± 0.008	−0.21 ± 0.02	−0.70 ± 0.10	1.00 ± 0.20	1.20 ± 0.10	1.70 ± 0.20
I172V	150 ± 8	0.010 ± 0.002	0.025 ± 0.007	−1.05 ± 0.10	−1.30 ± 0.30	−0.70 ± 0.10	−0.60 ± 0.05	−0.95 ± 0.40
L177A	330 ± 16	0.007 ± 0.002	0.022 ± 0.007	−1.23 ± 0.11	−1.40 ± 0.30	−1.30 ± 0.30	0.04 ± 0.06	−0.08 ± 0.22
V181A	170 ± 8	0.08 ± 0.02	0.027 ± 0.005	0.16 ± 0.01	−0.10 ± 0.02	0.50 ± 0.10	0.67 ± 0.05	1.22 ± 0.05
L190A	36 ± 3	0.80 ± 0.40	0.380 ± 0.220	1.54 ± 0.40	−0.30 ± 0.06	2.80 ± 0.40	0.45 ± 0.13	1.11 ± 0.10
T191S	260 ± 13	0.06 ± 0.01	0.021 ± 0.004	−0.03 ± 0.01	−0.17 ± 0.03	0.05 ± 0.02	*	*
L193A	120 ± 8	0.28 ± 0.05	0.060 ± 0.010	0.93 ± 0.05	0.24 ± 0.05	1.50 ± 0.40	0.37 ± 0.04	0.84 ± 0.03
V194A	38 ± 4	1.80 ± 0.30	0.150 ± 0.040	2.00 ± 0.40	0.70 ± 0.20	3.30 ± 0.80	0.38 ± 0.10	0.77 ± 0.04
V203A	190 ± 8	0.15 ± 0.03	0.026 ± 0.004	0.55 ± 0.03	0.30 ± 0.06	0.80 ± 0.10	0.33 ± 0.06	0.63 ± 0.08
T205S	230 ± 11	0.05 ± 0.01	0.023 ± 0.005	−0.14 ± 0.02	−0.33 ± 0.06	0.00 ± 0.01	*	*
L206A	210 ± 10	0.04 ± 0.01	0.022 ± 0.004	−0.21 ± 0.02	−0.36 ± 0.07	0.00 ± 0.01	*	*
T208S	270 ± 15	0.11 ± 0.02	0.040 ± 0.010	0.38 ± 0.03	−0.17 ± 0.03	0.44 ± 0.02	0.13 ± 0.14	1.39 ± 0.10
V209A	240 ± 12	0.18 ± 0.03	0.050 ± 0.010	0.65 ± 0.04	0.05 ± 0.01	0.78 ± 0.04	0.17 ± 0.08	0.93 ± 0.01
L210A	190 ± 10	0.06 ± 0.02	0.050 ± 0.010	0.05 ± 0.02	−0.60 ± 0.10	0.31 ± 0.02	*	*
L216A	460 ± 23	0.67 ± 0.12	0.024 ± 0.004	1.44 ± 0.13	1.20 ± 0.30	1.20 ± 0.10	−0.21 ± 0.12	−0.03 ± 0.21

* Variants reporting a ∆∆G_eq_ < 0.4 kcal mol^−1^ were excluded from the calculation of Φ‐values.

**FIGURE 3:**
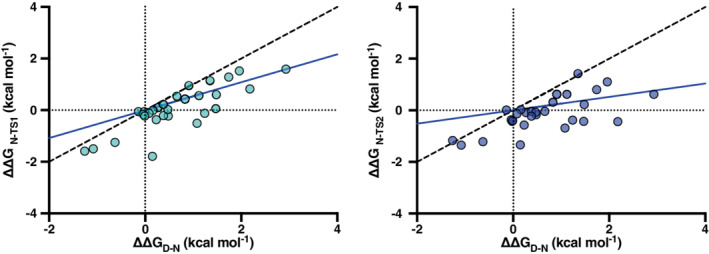
Linear Free Energy Relationship analysis for the early (left panel) and late (right panel) transition states. Continuous lines represent the best fit to a linear equation (see text for details), whereas broken black line represents a reference line with a slope = 1.

To further investigate the structural features of the early and late transition state of the folding reaction of the C‐SH2 domain we performed a Linear Free Energy Relationship (LFER) analysis.^16^ This method is based on relating the change in free energy observed in the transition states to the one calculated for the native state. The slope of the observed correlation, denoted as α value and ranging between 0 and 1, indicates the degree of native‐like structure in the transition state. By following this methodology, we performed a LFER analysis for both the transition states of the folding reaction of the C‐SH2 domain, and the corresponding LFER plots are reported in Figure 3. The LFER analysis returned a α_TS1_ = 0.46 ± 0.10 and α_TS2_ = 0.74 ± 0.10. Altogether, our results indicate that the C‐SH2 domain follows a stepwise folding mechanism with an extended native‐like late transition state.

### 2.2 Detecting possible Hammond effect on the transition states of C‐SH2

Global analysis of chevron plots obtained from several site‐directed variants of a given protein, or from different experimental conditions, is a very useful methodology to calculate reliable microscopic folding and unfolding rate constants.^15^ However, such a process relies on the assumption that the perturbation deriving from site‐directed mutagenesis or change in pH, ionic strength, etc., may have an effect only on the stability of the native state, without affecting the thermodynamics of the transition state(s). In particular, by sharing kinetic m‐values in the fitting process of chevron plots it is not possible to monitor possible changes in the transition states relative stability compared to the ground states, which, by following the Hammond postulate, could determine a movement of their position along the reaction pathway (the so‐called *Hammond effect*).^17^

**FIGURE 4:**
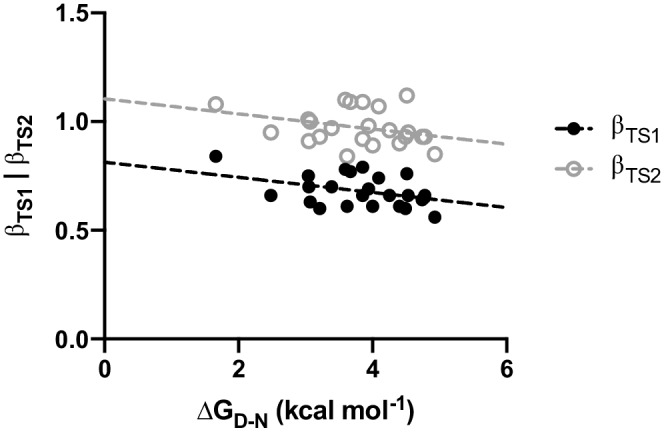
Hammond effect in the folding of the C‐SH2. Calculated β‐tanford values for the early transition state TS1 (in black) and for the late transition state TS2 (in gray) as a function of protein stability. Lines represent the best fit to a linear equation. β‐tanford values of the two transition states display clear dependences on overall stabilities highlighting the presence of Hammond effect (see text for details).

To obtain information about possible Hammond effects on the transition states of the C‐SH2 domain, we performed a new fit of the chevron plots reported in Figure 1 by removing constraints on all kinetic m‐values. Unfortunately, due to the data complexity, the analysis software (GraphPad Prism) could not compute a reliable fit (data not shown). Thus, we resorted to fit chevron plots by removing constraints only from m_f_ and m_u_ values, and we kept a shared m_part_ value for all data sets. Obtained kinetic and thermodynamic parameters are reported in Table S1. This approximation, which implies TS1 and TS2 positions on the reaction coordinate to be constant relatively to the high‐energy intermediate and to each other, allowed us to increase the complexity of our model and detect possible Hammond effects on TS1 and TS2 as function of the stability of the native state. The obtained β‐tanford values for TS1 and TS2 were plotted versus the thermodynamic stability of the corresponding site‐directed variant, and fitted with a linear equation (Figure 4). It is of interest to note that β_TS1_ and β_TS2_ values obtained display a clear dependence versus ∆G_D‐N_ values, with a negative slope. This result is compatible with a Hammond effect, highlighting that both transition states approach the native state on the reaction coordinate as the native state becomes less stable.^17^ To test the robustness of our results we resorted to globally analyze kinetic data by removing constraints from kinetic m_f_ value and monitoring the effect of the stability of the domain on the position of the early transition state on the reaction coordinate. We reported the results of the fitting process in Table S2, and the relative calculated β_TS1_ values as function of protein stability in Figure S1. Data were fitted with the same linear equation used to fit data reported in Figure 4, proving the robustness of the model proposed.

## 3 DISCUSSION

Although their fundamental role for the physiology of the cell and their involvement in several molecular pathways, only few experimental works characterized the folding properties of SH2 domains.^10^, ^13^, ^18^, ^19^, ^20^, ^21^ Understanding the determinants of thermodynamic stability as well as characterizing the folding pathway of a protein is of fundamental importance to depict the molecular basis of its biochemical function. Moreover, a structural characterization of the transition state(s) and of possible intermediates along the folding reaction allows to pinpoint potential aberrant misfolding events, that may result in protein misfunction. The analysis of Φ‐values represents a very powerful methodology to obtain such mechanistic information.

In the study of protein folding, it is particularly useful to compare the folding pathways of homologous proteins or domains. By analyzing the folding pathway of proteins belonging to the same family, sharing a similar three‐dimensional structure but displaying different primary structures, it is possible to characterize the role of sequence in determining folding and function, with the possibility to pinpoint interactions fundamental for proper folding and native state stability. To do so we resorted to compare kinetic and thermodynamic data obtained in a recent work for the N‐SH2 domain of SHP2 with data presented in this paper. The N‐SH2 domain is characterized by a three‐state folding mechanism implying the presence of an on‐pathway low‐energy intermediate. Interestingly the analysis of Φ‐values described a rather highly structured intermediate state, with native like contacts further locked in place in the late transition state. N‐SH2 domain kinetic data allowed us to calculate high β‐tanford values for both the early intermediate state and late transition state (β_I_ = 0.80 ± 0.03 β_TS2_ = 0.86 ± 0.04). These data well correlate with the highly native‐like structure depicted by the analysis of Φ‐values and describe a folding energy profile in which the intermediate state and late transition state are very close on the reaction coordinate. Interestingly, a comparison with the β‐tanford values obtained for the C‐SH2 domain (β_TS1_ = 0.61 ± 0.03 and β_TS2_ = 0.91 ± 0.04) indicate that the relative position of the early transition state of C‐SH2 and the intermediate state of N‐SH2 on the reaction coordinate are remarkably different. In fact, whilst the N‐SH2 domain appears to satisfy most of the native‐like contacts in the early intermediate state, the C‐SH2 domain shows a lower degree of native‐like structure, an aspect that may suggest the tendency to retain different degrees of residual structure in the denatured states of the two SH2 domains. In fact, it is worth noticing that for the SH2 domain of Src a considerable degree of residual structure in the denatured state could be detected.^18^ Since the denatured state in Φ‐value analysis is usually considered to be not affected in its free energy, it is tempting to assume that an undetected difference in the residual structure of the denatured states of the N‐SH2 or C‐SH2 domain might dictate the degree of native‐like structure characterizing early transient states of the two SH2 domains, possibly influencing the presence (and the relative stability) of intermediate states.

**FIGURE 5:**
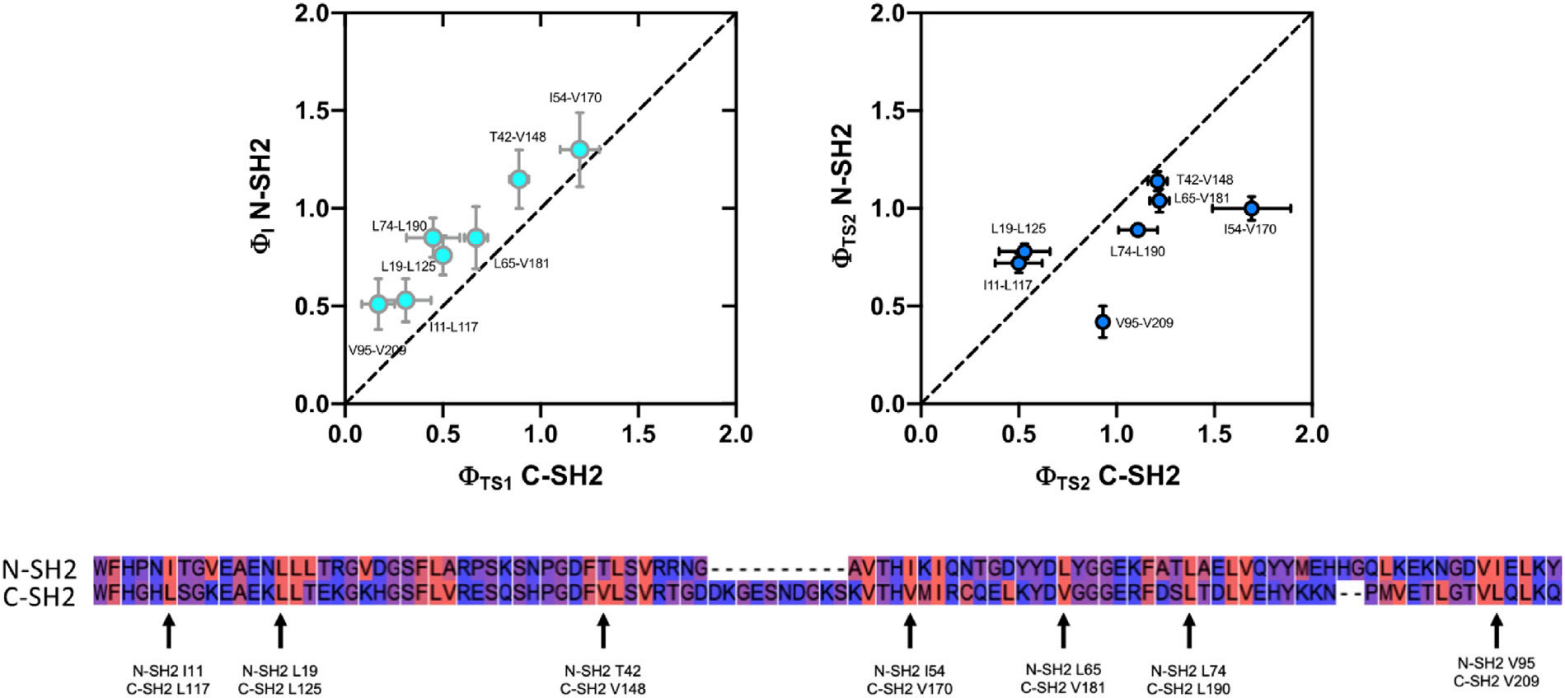
Φ vs Φ plots of early (top left panel) and late (top right panel) events of the folding reaction of N‐SH2 versus C‐SH2 domain. For each point, the relative position on the N‐SH2 and C‐SH2 domain is reported. Φ‐values of the early intermediate state of N‐SH2 are higher than the ones calculated for the TS1 of C‐SH2, indicating a conserved folding mechanism and an overall higher degree of native‐like structure in the early events of folding. Lines represent a linear equation with slope of 1. Bottom: alignment of the N‐SH2 and C‐SH2 domain performed with ClustalW online tool.

To further compare the folding pathway of the C‐SH2 domain with the N‐SH2 we resorted to perform a Φ vs Φ analysis for conserved residues between the two domains. Such analysis allows to determine the degree of similarities in native‐like structure between probed states, providing useful information about whether folding pathways are conserved in homologous proteins.^22^ To obtain an informative Φ vs Φ plot, a sequence alignment of the two domains is mandatory. In fact, in such analysis, only residues in the same structural position in the two proteins can be compared. To do so, we performed a sequence alignment between the N‐SH2 and C‐SH2 domain of SHP2 by using the ClustalW online tool (https://www.genome.jp/tools-bin/clustalw). In Figure 5 we show Φ vs Φ plots of early and late events of the folding reaction of N‐SH2 vs C‐SH2 domain and we reported the alignment result, highlighting those residues for which a comparison of Φ‐values was possible. For the early events of folding, albeit the clear linear correlation between the Φ‐values of the two domains, Φ‐values of N‐SH2 are higher than the ones calculated for C‐SH2, indicating a conserved folding mechanism with a higher degree of native‐like structure for the N‐SH2 domain. The analysis of the late events of folding, on the other hand, appears less clear, due to a more scattered correlation between Φ‐values. Interestingly, a comparison of the change in stability of the early and late events of folding, as well as of the native state of C‐SH2 and N‐SH2 domains upon mutation (Figure S2) resemble what is reported in Figure 5, further supporting the validity of our model and the hypothesis of a generally conserved folding mechanism between the C‐SH2 and N‐SH2 domain.

The analysis of Φ‐values of C‐SH2 domain show the presence of anomalous Φ‐values in both early and late events of folding, in correspondence of residues V170 and I172. Structural explanation of unusual Φ‐values demands careful analysis, given the nature of Φ‐values as thermodynamic measures of native‐like structure. The presence of Φ‐values higher than 1 and/or lower than 0 is, in fact, usually interpreted as the presence of non‐native interactions, which may lead to misfolding events. It is worth noticing that, as reported in Table 1, there are other positions characterized by an unusual Φ‐value. However, since those mutations caused a minor change in activation and/or equilibrium free energies that may jeopardize Φ‐value calculation, we considered those Φ‐values as low (close to 0) or high (close to 1), to avoid misinterpretation of thermodynamic parameters, and reported them accordingly in Figure 2. Interestingly, both V170 and I172 residues are physically located close to the binding pocket of the C‐SH2 domain. Thus, it is not surprising that perturbation occurring in the functional site of the protein lead to the occurrence of transient misfolding events. Whilst, in fact, globular proteins are generally characterized by a status of minimal frustration,^23^, ^24^ that is, residues have been selected by evolution to best fulfill the interactions needed for the protein to fold, the contrasting demand between folding and function leads to the presence of residues that are sub‐optimal for folding in the functional sites of proteins.^23^, ^24^, ^25^, ^26^ Given these premises, we conclude that the unusual Φ‐values measured for V170 and I172 highlight these residues to be part of a frustration pattern relative to the functional binding site of the C‐SH2 domain.

In summary, in this work we provide a comprehensive characterization of the folding mechanism of the C‐terminal SH2 domain of SHP2 protein, with a detailed characterization of early and late events of folding. These kinds of analysis is of particular interest to understand the determinants of stability of functional proteins and domains, such as SH2. In fact, albeit deep learning and artificial intelligence (AI) approaches, as for example AlphaFold^27^ and RoseTTA,^28^ are currently paving the way into a better and better determination of three‐dimentional structure of proteins starting only from their aminoacidic sequences, the characterization of the mechanism by how the native functional shape is acquired still strongly relies on experimental data. Moreover, given the enormous number of physiological pathways that are regulated by protein–protein interactions mediated by these types of domains, determining such mechanisms allows to pinpoint possible misfolding events that are, until now, elusive for AIs. Data reported in this study, in fact, show how residues structurally located in the functional portion of the domain to display transient misfolding, as reported by their unusual values of Φ. The comparison of kinetic and thermodynamic folding data with the ones obtained for the N‐SH2 domain suggest a conserved complex folding mechanism with the formation of at least one intermediate along the folding reaction. However, the relative stability of such intermediate appears to be highly sequence dependent. Under this light, we cannot exclude that the folding mechanism of SH2 domains showing a two‐state behavior^18^, ^20^ may involve intermediate state(s) too unstable to be experimentally detected. Moreover, by systematically comparing Φ‐values obtained for conserved residues between the N‐SH2 and C‐SH2 domains we could highlight a conserved and robust mechanism, in both early and late events of folding.

## 4 MATERIALS AND METHODS

### 4.1 Protein expression and purification

C‐SH2 domain in its wild‐type variant and all the site‐directed mutants produced were purified as described previously.^13^ Site‐directed mutagenesis was performed using the QuikChange mutagenesis kit (Agilent Technologies Inc., Santa Clara, CA), accordingly to manufacturer instructions.

### 4.2 Kinetic (un)folding experiments

Kinetic (un)folding experiments were performed on an Applied Photophysics Pi‐star 180 stopped‐flow apparatus. Samples were excited at 280 nm and the change of fluorescence emission was recorded by using a 360 nm cutoff glass filter. The experiments were performed at 25°C in buffer Tris HCl 50 mM, DTT 2 mM, pH 8.0, by using urea as denaturant agent. For each denaturant concentration, at least five individual traces were averaged. The final protein concentration was typically 2 μM.

### 4.3 Data analysis

In a two‐state scenario the logarithm of the folding rate constants follows a linear dependence as function of [UREA], with the following equation used to fit a typical two‐state chevron plot

$$k_{\mathrm{obs}}=\log\;\left(k_{f}^{0}\exp\left(-m_{f}\left[\text{UREA}\right]/\mathrm{RT}\right)+k_{u}^{0}\exp\left(m_{u}\left[\text{UREA}\right]/\mathrm{RT}\right)\right)$$

The parameters m_f_ and m_u_ represent the slopes of the dependence of k_f_ and k_u_ as function of [UREA]. In the case of the C‐SH2 domain, a kink in the unfolding arm of the chevron plot is clearly appreciable. The three‐state model used to describe kinetic data implied the presence of an on‐pathway high‐energy intermediate

$$k_{\mathrm{obs}}=k_{f}^{0}\exp\left(-m_{f}\left[\text{UREA}\right]/\mathrm{RT}\right)+\frac{k_{u}^{0}\exp\left(m_{u}\left[\text{UREA}\right]/\mathrm{RT}\right)}{1+K_{\text{part}}\exp\left(m_{\text{part}}\left[\text{UREA}\right]/\mathrm{RT}\right)}$$

with K_part_ representing the partitioning constant between the early (TS1) and late (TS2) transition state, and m_part_ is the associated kinetic m‐value.

β‐tanford values for TS1 and TS2 were calculated as follows

$$\beta_{\text{TS1}}=m_{D-\text{TS1}}/m_{D-N}$$

and

$$\beta_{\text{TS2}}=m_{D-\text{TS2}}/m_{D-N}=1-\left[\left(m_{\mathrm{TS}1-N}-m_{\text{part}}\right)/m_{D-N}\right]$$

Φ‐values for TS1 were calculated using the following equation

$$\Phi_{\text{TS1}}=\text{∆}∆G_{D-\text{TS1}}/\text{∆}∆G_{D-N}$$

where

$$∆∆G_{D-\mathrm{TS}1}={∆G}_{D-\mathrm{TS}1}^{\mathrm{wt}}-{∆G}_{D-\mathrm{TS}1}^{\mathrm{mut}}=\mathrm{RT}\;\ln\;\left(k_{f}^{\mathrm{wt}}-k_{f}^{\mathrm{mut}}\right)$$

$$∆∆G_{D-N}={∆G}_{D-N}^{\mathrm{wt}}-{∆G}_{D-N}^{\mathrm{mut}}$$

Φ‐values for TS2 were calculated as

$$\Phi_{\text{TS2}}=1-\left(\text{∆}∆G_{\mathrm{TS}2-N}/\text{∆}∆G_{D-N}\right)$$

where

$$∆∆G_{\mathrm{TS}2-N}={∆G}_{\mathrm{TS}2-N}^{\mathrm{wt}}-{∆G}_{\mathrm{TS}2-N}^{\mathrm{mut}}=\mathrm{RT}\;\ln\;\left(k_{u}^{\mathrm{mut}}/k_{\text{part}}^{\mathrm{mut}}-k_{u}^{\mathrm{wt}}/k_{\text{part}}^{\mathrm{wt}}\right)$$

## AUTHOR CONTRIBUTIONS

**Angelo Toto:** Conceptualization (lead); formal analysis (lead); investigation (lead); writing – original draft (lead); writing – review and editing (lead). **Francesca Malagrinò:** Investigation (equal); methodology (equal); writing – review and editing (supporting). **Caterina Nardella:** Investigation (equal); methodology (equal); writing – review and editing (supporting). **Valeria Pennacchietti:** Investigation (equal); methodology (equal); writing – review and editing (supporting). **Livia Pagano:** Investigation (equal); methodology (equal); writing – review and editing (supporting). **Daniele Santorelli:** Investigation (equal); methodology (equal); writing – review and editing (supporting). **Awa Diop:** Investigation (equal); methodology (equal); writing – review and editing (supporting). **Stefano Gianni:** Conceptualization (lead); resources (lead); writing – review and editing (lead).

## Supporting information


**Table S1.** Folding and thermodynamic parameters of C‐SH2 domain variants obtained from a global fitting process sharing kinetic m_part_ = 0.45 ± 0.02 kcal mol^−1^ M^−1^. L102A, L117A, T153S, T168S, V170A, L177A, and L206A were excluded from analysis due to high error in fitting process.
**Table S2**. Folding and thermodynamic parameters of C‐SH2 domain variants obtained from a global fitting process sharing kinetic m_u_ = 0.52 ± 0.03 kcal mol^−1^ M^−1^ and m_part_ = 0.45 ± 0.02 kcal mol^−1^ M^−1^
**Figure S1**. Calculated β‐tanford values for the early transition state TS1 (in black) and for the late transition state TS2 (in gray) as a function of protein stability, by removing constraints only for the mf value in global fitting process (see text for details).
**Figure S2**. Comparison of the effect on change in free energy of early events of folding (left panel), late transition states (central panel) and native states (right panel) for the C‐SH2 and N‐SH2 domain.
