# Supplementary material for: Characterization of early and late transition states of the folding pathway of a SH2 domain
Source: Protein Sci. 2022 May 17;31(6):e4332. doi: 10.1002/pro.4332 (PMC9112803; doi:10.1002/pro.4332)
Supplement: Supplementary file 1 — Table S1. Folding and thermodynamic parameters of C‐SH2 domain variants obtained from a global fitting process sharing kinetic mpart = 0.45 ± 0.02 kcal mol−1 M−1. L102A, L117A, T153S, T168S, V170A, L177A, and L206A were excluded from analysis due to high error in fitting process. Table S2. Folding and thermodynamic parameters of C‐SH2 domain variants obtained from a global fitting process sharing kinetic mu = 0.52 ± 0.03 kcal mol−1 M−1 and mpart = 0.45 ± 0.02 kcal mol−1 M−1 Figure S1. Calculated β‐tanford values for the early transition state TS1 (in black) and for the late transition state TS2 (in gray) as a function of protein stability, by removing constraints only for the mf value in global fitting process (see text for details). Figure S2. Comparison of the effect on change in free energy of early events of folding (left panel), late transition states (central panel) and native states (right panel) for the C‐SH2 and N‐SH2 domain. [file PRO-31-e4332-s001.docx]

|  | k_f_ (s^-1^) | k_u_ (s^-1^) | k_part_ | m_f_  (kcal mol^-1^ M^-1^) | m_u_  (kcal mol^-1^ M^-1^) | ∆∆G_TS1_  (kcal mol^-1^) | ∆∆G_TS2_  (kcal mol^-1^) | ∆∆G_eq_  (kcal mol^-1^) |
| --- | --- | --- | --- | --- | --- | --- | --- | --- |
| **wt** | 370 ± 30 | 0.11 ± 0.09 | 0.021 ± 0.004 | 1.03 ± 0.03 | 0.56 ± 0.20 |  |  |  |
| **L102A** |  |  |  |  |  |  |  |  |
| **A105G** | 352 ± 22 | 0.12 ± 0.01 | 0.039 ± 0.003 | 0.98 ± 0.02 | 0.55 ± 0.08 | 0.00 ± 0.02 | -0.35 ± 0.08 | 0.04 ± 0.01 |
| **T108S** | 325 ± 24 | 0.48 ± 0.09 | 0.001 ± 0.001 | 1.18 ± 0.03 | 0.31 ± 0.03 | 0.85 ± 0.10 | 2.90 ± 0.60 | 0.90 ± 0.20 |
| **L117A** |  |  |  |  |  |  |  |  |
| **A122G** | 111 ± 21 | 0.06 ± 0.08 | 0.029 ± 0.010 | 0.94 ± 0.07 | 0.60 ± 0.06 | -0.34 ± 0.03 | -0.53 ± 0.08 | 0.37 ± 0.08 |
| **L125A** | 165 ± 19 | 0.36 ± 0.11 | 0.244 ± 0.120 | 1.18 ± 0.06 | 0.74 ± 0.02 | 0.67 ± 0.10 | -0.76 ± 0.10 | 1.15 ± 0.60 |
| **L126A** | 405 ± 40 | 0.60 ± 0.09 | 0.045 ± 0.014 | 1.12 ± 0.04 | 0.57 ± 0.08 | 0.97 ± 0.12 | 0.53 ± 0.10 | 0.93 ± 0.10 |
| **T127S** | 284 ± 46 | 0.13 ± 0.19 | 0.014 ± 0.004 | 0.99 ± 0.07 | 0.52 ± 0.03 | 0.08 ± 0.01 | 0.34 ± 0.08 | 0.25 ± 0.03 |
| **L136A** | 209 ± 35 | 1.18 ± 0.10 | 0.117 ± 0.041 | 1.44 ± 0.10 | 0.62 ± 0.02 | 1.40 ± 0.30 | 0.37 ± 0.04 | 1.70 ± 0.40 |
| **V137A** | 14 ± 1 | 0.01 ± 0.03 | 0.036 ± 0.033 | 0.88 ± 0.03 | 0.68 ± 0.12 | -2.11 ± 0.20 | -2.41 ± 0.60 | -0.16 ± 0.08 |
| **V148A** | 38 ± 3 | 0.22 ± 0.03 | 0.012 ± 0.004 | 1.25 ± 0.03 | 0.42 ± 0.03 | 0.38 ± 0.04 | 0.75 ± 0.10 | 1.70 ± 0.30 |
| **L149A** | 38 ± 12 | 0.12 ± 0.13 | 0.018 ± 0.005 | 1.15 ± 0.30 | 0.49 ± 0.03 | 0.04 ± 0.01 | 0.15 ± 0.02 | 1.40 ± 0.10 |
| **T153S** |  |  |  |  |  |  |  |  |
| **T168S** |  |  |  |  |  |  |  |  |
| **V170A** |  |  |  |  |  |  |  |  |
| **I172V** | 144 ± 11 | 0.07 ± 0.03 | 0.001 ± 0.001 | 0.93 ± 0.02 | 0.30 ± 0.03 | -0.30 ± 0.03 | 2.14 ± 0.40 | 0.26 ± 0.05 |
| **L177A** |  |  |  |  |  |  |  |  |
| **V181A** | 209 ± 27 | 0.48 ± 0.14 | 0.001 ± 0.001 | 1.06 ± 0.05 | 0.30 ± 0.04 | 0.84 ± 0.10 | 3.30 ± 0.80 | 1.20 ± 0.20 |
| **L190A** | 38 ± 4 | 0.56 ± 0.05 | 0.112 ± 0.034 | 0.96 ± 0.07 | 0.51 ± 0.02 | 0.94 ± 0.10 | -0.04 ± 0.01 | 2.30 ± 0.40 |
| **T191S** | 298 ± 26 | 0.29 ± 0.08 | 0.002 ± 0.002 | 1.01 ± 0.03 | 0.35 ± 0.04 | 0.55 ± 0.06 | 2.10 ± 0.70 | 0.68 ± 0.10 |
| **L193A** | 84 ± 8 | 0.36 ± 0.09 | 0.032 ± 0.003 | 0.79 ± 0.04 | 0.53 ± 0.09 | 0.68 ± 0.07 | 0.44 ± 0.10 | 1.60 ± 0.50 |
| **V194A** | 67 ± 19 | 4.00 ± 0.40 | 0.006 ± 0.002 | 1.59 ± 0.24 | 0.30 ± 0.02 | 2.10 ± 0.30 | 2.81 ± 0.40 | 3.10 ± 0.40 |
| **V203A** | 191 ± 21 | 0.22 ± 0.04 | 0.043 ± 0.005 | 0.95 ± 0.05 | 0.60 ± 0.11 | 0.37 ± 0.03 | -0.05 ± 0.01 | 0.77 ± 0.07 |
| **T205S** | 309 ± 19 | 0.09 ± 0.03 | 0.028 ± 0.003 | 1.05 ± 0.02 | 0.55 ± 0.11 | -0.12 ± 0.01 | -0.28 ± 0.05 | 0.00 ± 0.05 |
| **L206A** |  |  |  |  |  |  |  |  |
| **T208S** | 291 ± 25 | 0.22 ± 0.11 | 0.025 ± 0.003 | 0.97 ± 0.03 | 0.49 ± 0.05 | 0.38 ± 0.04 | 0.29 ± 0.06 | 0.53 ± 0.08 |
| **V209A** | 312 ± 34 | 0.40 ± 0.26 | 0.025 ± 0.005 | 1.06 ± 0.05 | 0.47 ± 0.05 | 0.73 ± 0.10 | 0.63 ± 0.12 | 0.84 ± 0.10 |
| **L210A** | 135 ± 17 | 0.07 ± 0.06 | 0.025 ± 0.005 | 0.80 ± 0.04 | 0.54 ± 0.05 | -0.32 ± 0.03 | -0.41 ± 0.08 | 0.28 ± 0.04 |
| **L216A** | 278 ± 30 | 1.50 ± 0.60 | 0.003 ± 0.003 | 0.73 ± 0.05 | 0.43 ± 0.06 | 1.50 ± 0.20 | 2.65 ± 0.60 | 1.70 ± 0.20 |

Table S1 - Folding and thermodynamic parameters of C-SH2 domain variants obtained from a global fitting process sharing kinetic m_part_ = 0.45 ± 0.02 kcal mol^-1^ M^-1^. L102A, L117A, T153S, T168S, V170A, L177A, L206A were excluded from analysis due to high error in fitting process.

|  | k_f_ (s^-1^) | k_u_ (s^-1^) | k_part_ | m_f_  (kcal mol^-1^ M^-1^) | ∆∆G_TS1_  (kcal mol^-1^) | ∆∆G_TS2_  (kcal mol^-1^) | ∆∆G_eq_  (kcal mol^-1^) |
| --- | --- | --- | --- | --- | --- | --- | --- |
| **wt** | 375 ± 30 | 0.13 ± 0.02 | 0.013 ± 0.003 | 1.03 ± 0.02 |  |  |  |
| **L102A** | 427 ± 38 | 0.12 ± 0.02 | 0.011 ± 0.003 | 1.10 ± 0.03 | -0.06 ± 0.01 | 0.01 ± 0.01 | -0.14 ± 0.01 |
| **A105G** | 350 ± 28 | 0.12 ± 0.02 | 0.024 ± 0.007 | 0.97 ± 0.03 | -0.06 ± 0.01 | -0.43 ± 0.06 | -0.02 ± 0.01 |
| **T108S** | 282 ± 26 | 0.16 ± 0.02 | 0.018 ± 0.005 | 1.10 ± 0.03 | 0.10 ± 0.01 | -0.09 ± 0.01 | 0.27 ± 0.03 |
| **L117A** | 178 ± 40 | 1.77 ± 0.03 | 0.026 ± 0.008 | 1.42 ± 0.14 | 1.50 ± 0.15 | 1.10 ± 0.10 | 1.90 ± 0.20 |
| **A122G** | 112 ± 10 | 0.09 ± 0.02 | 0.012 ± 0.003 | 0.95 ± 0.03 | -0.23 ± 0.02 | -0.17 ± 0.02 | 0.48 ± 0.5 |
| **L125A** | 146 ± 17 | 0.35 ± 0.02 | 0.012 ± 0.003 | 1.11 ± 0.05 | 0.56 ± 0.06 | 0.62 ± 0.06 | 1.10 ± 0.10 |
| **L126A** | 400 ± 40 | 0.68 ± 0.08 | 0.023 ± 0.006 | 1.12 ± 0.05 | 0.95 ± 0.09 | 0.62 ± 0.06 | 0.91 ± 0.10 |
| **T127S** | 283 ± 30 | 0.13 ± 0.02 | 0.012 ± 0.003 | 0.99 ± 0.03 | -0.01 ± 0.01 | 0.02 ± 0.01 | 0.16 ± 0.01 |
| **L136A** | 174 ± 46 | 1.18 ± 0.12 | 0.029 ± 0.008 | 1.32 ± 0.15 | 1.30 ± 0.10 | 0.80 ± 0.10 | 1.70 ± 0.20 |
| **V137A** | 14 ± 1 | 0.010 ± 0.001 | 0.006 ± 0.003 | 0.89 ± 0.03 | -1.80 ± 0.20 | -1.30 ± 0.40 | 0.15 ± 0.02 |
| **V148A** | 34 ± 5 | 0.15 ± 0.01 | 0.030 ± 0.008 | 1.18 ± 0.07 | 0.06 ± 0.01 | -0.43 ± 0.04 | 1.50 ± 0.20 |
| **L149A** | 37 ± 5 | 0.11 ± 0.07 | 0.020 ± 0.005 | 1.13 ± 0.07 | -0.12 ± 0.01 | -0.38 ± 0.04 | 1.20 ± 0.10 |
| **T153S** | 183 ± 15 | 0.010 ± 0.001 | 0.010 ± 0.003 | 0.83 ± 0.02 | -1.50 ± 0.20 | -1.40 ± 0.20 | -1.10 ± 0.10 |
| **T168S** | 136 ± 13 | 0.09 ± 0.01 | 0.010 ± 0.003 | 0.95 ± 0.03 | -0.21 ± 0.02 | -0.05 ± 0.01 | 0.38 ± 0.05 |
| **V170A** | 25 ± 3 | 0.06 ± 0.01 | 0.018 ± 0.005 | 0.79 ± 0.04 | -0.51 ± 0.05 | -0.69 ± 0.07 | 1.08 ± 0.10 |
| **I172V** | 130 ± 11 | 0.020 ± 0.002 | 0.012 ± 0.004 | 0.89 ± 0.03 | -1.25 ± 0.10 | -1.20 ± 0.20 | -0.63 ± 0.07 |
| **L177A** | 210 ± 18 | 0.010 ± 0.002 | 0.006 ± 0.003 | 0.82 ± 0.02 | -1.60 ± 0.20 | -1.20 ± 0.20 | -1.26 ± 0.03 |
| **V181A** | 176 ± 17 | 0.14 ± 0.02 | 0.015 ± 0.004 | 0.96 ± 0.02 | 0.02 ± 0.01 | -0.08 ± 0.01 | 0.47 ± 0.05 |
| **L190A** | 37 ± 10 | 0.54 ± 0.10 | 0.110 ± 0.050 | 0.96 ± 0.15 | 0.82 ± 0.08 | -0.44 ± 0.05 | 2.20 ± 0.20 |
| **T191S** | 270 ± 26 | 0.11 ± 0.02 | 0.013 ± 0.004 | 0.96 ± 0.03 | -0.12 ± 0.01 | -0.14 ± 0.01 | 0.08 ± 0.01 |
| **L193A** | 84 ± 11 | 0.37 ± 0.05 | 0.025 ± 0.007 | 0.79 ± 0.06 | 0.60 ± 0.06 | 0.22 ± 0.02 | 1.50 ± 0.30 |
| **V194A** | 39 ± 11 | 2.00 ± 0.20 | 0.070 ± 0.020 | 1.00 ± 0.20 | 1.60 ± 0.20 | 0.62 ± 0.06 | 2.90 ± 0.30 |
| **V203A** | 191 ± 18 | 0.28 ± 0.04 | 0.016 ± 0.004 | 0.96 ± 0.03 | 0.43 ± 0.03 | 0.31 ± 0.03 | 0.83 ± 0.09 |
| **T205S** | 310 ± 30 | 0.10 ± 0.02 | 0.018 ± 0.005 | 1.04 ± 0.03 | -0.16 ± 0.02 | -0.37 ± 0.04 | -0.04 ± 0.01 |
| **L206A** | 260 ± 24 | 0.09 ± 0.01 | 0.017 ± 0.005 | 1.03 ± 0.03 | -0.23 ± 0.02 | -0.40 ± 0.04 | -0.01 ± 0.01 |
| **T208S** | 290 ± 35 | 0.20 ± 0.02 | 0.028 ± 0.008 | 0.97 ± 0.04 | 0.22 ± 0.02 | -0.23 ± 0.02 | 0.38 ± 0.04 |
| **V209A** | 310 ± 32 | 0.34 ± 0.04 | 0.040 ± 0.010 | 1.06 ± 0.04 | 0.54 ± 0.06 | -0.04 ± 0.01 | 0.66 ± 0.07 |
| **L210A** | 135 ± 12 | 0.07 ± 0.01 | 0.018 ± 0.005 | 0.80 ± 0.03 | -0.37 ± 0.04 | -0.57 ± 0.06 | 0.23 ± 0.03 |
| **L216A** | 264 ± 24 | 0.94 ± 0.15 | 0.008 ± 0.002 | 0.69 ± 0.04 | 1.10 ± 0.10 | 1.40 ± 0.10 | 1.40 ± 0.20 |

Table S2 - Folding and thermodynamic parameters of C-SH2 domain variants obtained from a global fitting process sharing kinetic m_u_ = 0.52 ± 0.03 kcal mol^-1^ M^-1^ and m_part_ = 0.45 ± 0.02 kcal mol^-1^ M^-1^

FIG S1 - Calculated β-tanford values for the early transition state TS1 (in black) and for the late transition state TS2 (in gray) as a function of protein stability, by removing constraints only for the mf value in global fitting process (see text for details).

FIG S2 - Comparison of the effect on change in free energy of early events of folding (left panel), late transition states (central panel) and native states (right panel) for the C-SH2 and N-SH2 domain.
